# Within-host mechanisms of immune regulation explain the contrasting dynamics of two helminth species in both single and dual infections

**DOI:** 10.1371/journal.pcbi.1008438

**Published:** 2020-11-23

**Authors:** Chiara Vanalli, Lorenzo Mari, Lorenzo Righetto, Renato Casagrandi, Marino Gatto, Isabella M. Cattadori

**Affiliations:** 1 Center for Infectious Disease Dynamics and Department of Biology, The Pennsylvania State University, University Park, Pennsylvania, USA; 2 Dipartimento di Elettronica, Informazione e Bioingegneria, Politecnico di Milano, Milano, Italy; Ecole Polytechnique Fédérale de Lausanne, SWITZERLAND

## Abstract

Variation in the intensity and duration of infections is often driven by variation in the network and strength of host immune responses. While many of the immune mechanisms and components are known for parasitic helminths, how these relationships change from single to multiple infections and impact helminth dynamics remains largely unclear. Here, we used laboratory data from a rabbit-helminth system and developed a within-host model of infection to investigate different scenarios of immune regulation in rabbits infected with one or two helminth species. Model selection suggests that the immunological pathways activated against *Trichostrongylus retortaeformis* and *Graphidium strigosum* are similar. However, differences in the strength of these immune signals lead to the contrasting dynamics of infections, where the first parasite is rapidly cleared and the latter persists with high intensities. In addition to the reactions identified in single infections, rabbits with both helminths also activate new pathways that asymmetrically affect the dynamics of the two species. These new signals alter the intensities but not the general trend of the infections. The type of interactions described can be expected in many other host-helminth systems. Our immune framework is flexible enough to capture different mechanisms and their complexity, and provides essential insights to the understanding of multi-helminth infections.

## Author summary

Hosts infected with parasites have developed complex immune strategies to regulate infection severity, however, these strategies are not always successful to confer long term protection. Here, we examine the immune reactions of hosts infected with one or two gastrointestinal parasites, using a rabbit-helminth system and a modeling approach to laboratory experiments. We found that similar immune interactions operate for the two parasites, however, changes in the strength of the relationships lead to contrasting dynamics of infections, where one parasite is quickly removed while the other shows no evidence of control. For hosts infected with both parasites we also found the activation of new immune reactions that asymmetrically affect the dynamics of the two parasites. While we observed changes in parasite intensity the general trends are conserved. Understanding how the immune reaction modulates host-parasite interactions can help to explain the often large variation in the host response to infections in natural systems.

## Introduction

Population-level processes of infection are strongly affected by the way parasites interact with the host immune response. These responses are complex, and involve components and functions that are time and space dependent, whilst targeting specific attributes and phases of the infecting parasite (both macro- and micro-parasites). For hosts that are infected by more than one parasite species the strength of these reactions is predicted to change when compared to hosts with single infections. On the one hand, we could expect that the immune mechanisms are fundamentally conserved but their magnitude varies based on the properties of the co-infecting parasites and the history of host previous infections [1]. On the other hand, the network of interactions could be altered, such that new immune functions could be activated or suppressed, with consequences that are not fully predictable from basic knowledge on single infections [2]. Disentangling the critical mechanisms and their impact on each parasite species is challenging because of the often limited information on the immune network and the interactions with the co-infecting parasites.

Within-host mathematical models provide a tool to test some of these hypotheses by offering a mechanistic understanding of the host-parasite relationships through a simplified description of the immune reactions and constituents that affect the dynamics of infection. These frameworks have been primarily built on single infections and follow a phenomenological approach based on current knowledge of the biology and immunology of the target parasite-host system [3–8]. For example, conceptual models have been developed to explore the dynamics of effectors, such as cytokines, T-cells or antibodies, during the initial phase of parasite population expansion [7, 9–12] or the later stage of parasite killing and removal [13, 14], or by considering both phases [13, 15–17]. The evidence that hosts are often infected by more than one species has shifted the attention towards the contribution of immunological and ecological drivers to the interactions between parasites and the consequences for their dynamics and evolution. The general approach is to investigate the target parasite and to assume a functional response from the presence of the second species, either through immune mediated interactions, such as cross-reaction [18–20], or ecological processes, for example, interference competition for resources [21, 22]. For co-infecting helminths, within-host models have primarily investigated the ecological mechanisms of species interaction, often focusing on direct competitions in the same organ [23–25]. Models that explicitly address the mediated role of host immunity are rare [26–28], which contrasts with studies on helminth immunology, where many of the fundamental mechanisms of host-parasite interaction have been well characterized [29, 30].

Parasitic helminths usually stimulate a type 2 immune reaction that involves cytokines and transcription factors like IL4, IL5, IL13 and GATA3, bone marrow produced eosinophils and B-cell generated antibodies, such as IgA and IgE [31]. Understanding how the network of these and other immune components impact each co-infecting species, including how these relationships differ from single infections, can contribute to explain the often large variation in disease severity and parasite transmission commonly observed among hosts.

Here, we present a formalism for the within-host immuno-dynamics of single and dual infections using a helminth-rabbit system. Our model is sufficiently general to capture the critical immune constraints to each helminth species, while allowing for flexibility in the number of immune variables and interactions that can be examined. The framework is independently applied to single infections of *Trichostrongylus retortaeformis* and *Graphidium strigosum*, two common gastrointestinal helminths of the European rabbit (*Oryctolagus cuniculus*), and then adapted to examine the case of rabbits with both infections. Different hypotheses on the mechanisms of host-parasite and parasite-parasite interactions are tested. Model parameterization is based on available laboratory experiments where host immunity and helminth data were collected at fixed time intervals. Simulations from the best model indicate that the fundamental immune reactions are conserved against the two helminths, however, changes in their strength lead to contrasting dynamics of infection. Helminths primarily interact via cross-stimulation, where the immune response to the first parasite species is also stimulated by the presence of the second species. These cross-interactions are asymmetric and further contribute to the variation in infection observed, both within and between helminth species.

## Materials and methods

### The system and experimental infections

**Fig 1:**
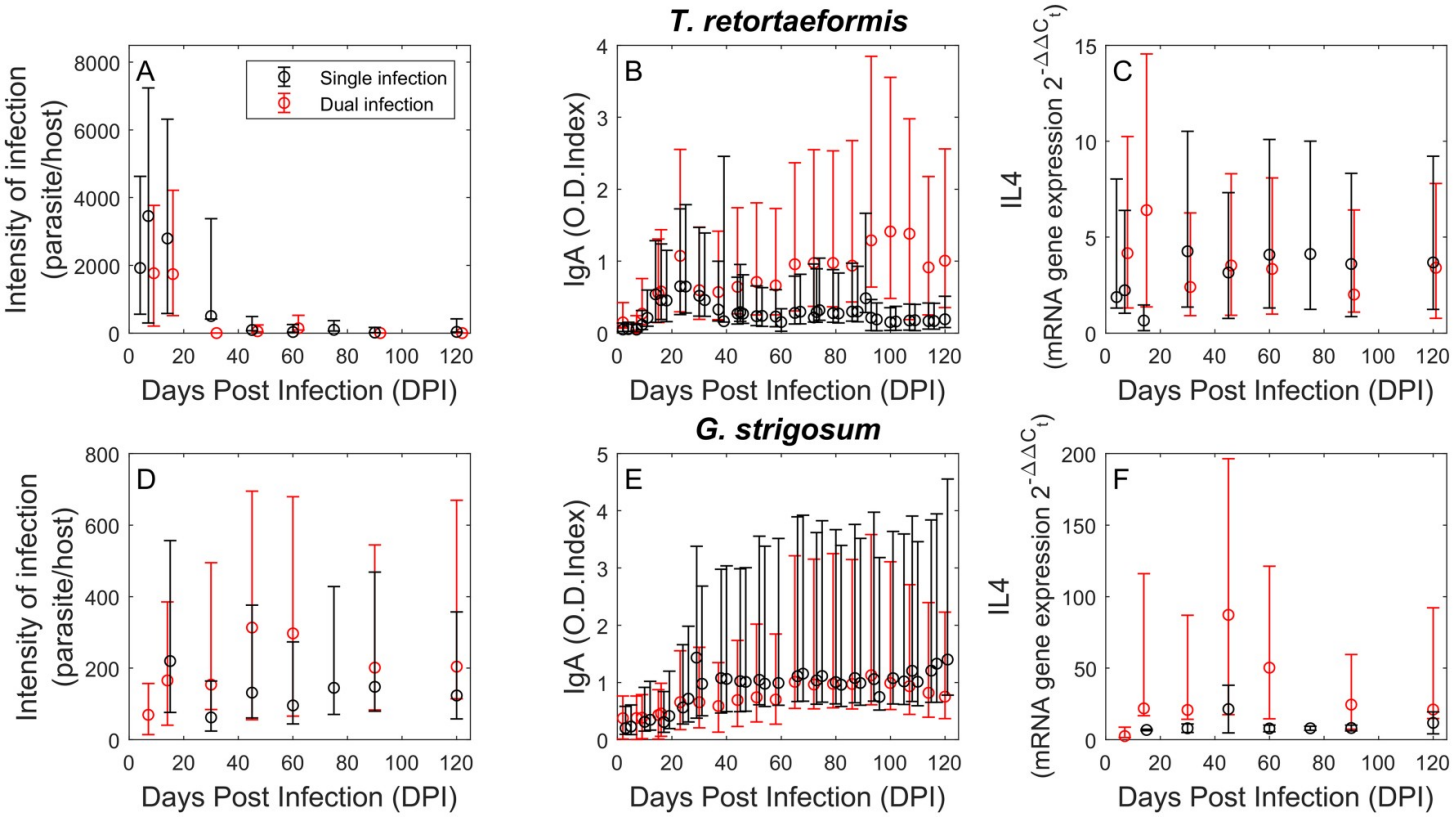
Experimental data of single and dual infections. Intensity of infection (A and D), specific IgA antibody response estimated using adult worms (B) or excretory-secretory (ES) products from adult worms as the source of antigen (E), and IL4 cytokine gene expression (C and F) during single (black) and dual (red) infections of *T. retortaeformis* (A-C) and *G. strigosum* (D-F). Geometric means and relative dispersions (calculated as product/ratio between the geometric mean and the geometric standard deviation) are presented.

In endemic areas *Trichostrongylus retortaeformis* (TR) and *Graphidium strigosum* (GS) cause chronic infections in European rabbits. Infections occur by ingestion of infective larvae that develop into adults in the gastrointestinal tract; *T. retortaeformis* colonises the small intestine, mainly the duodenum, while *G. strigosum* inhabits the stomach, primarily the fundus. For the purpose of this study, we used data from laboratory experiments available from Murphy et al. [31, 32]. Briefly, rabbits were infected with a single inoculum of 5 ml of water with either 5500 *T. retortaeformis* or 650 *G. strigosum* infective larvae, or both for rabbits with dual infections; control hosts received only water. The dynamics of infection and host immune response were then followed for 120 days by sacrificing four infected and two control animals at fixed time points, chosen to represent important steps during parasite development and related immune reaction. These experiments showed that following the single inoculum of either one or both helminths, *T. retortaeformis* was successfully reduced and in many animals removed from the small intestine, while *G. strigosum* maintained high intensities throughout the trials (Fig 1A and 1D). In both single and dual infections rabbits developed an anti-inflammatory type 2 reaction, which involved the production of cytokine IL4, species-specific antibodies IgA and IgG and eosinophils [31, 32].

Our within-host model of immune regulation was developed using parasite intensities and IL4 gene expression quantified in the duodenum and fundus of rabbits available at sacrificing time (Fig 1A, 1C, 1D and 1F); species-specific IgA was measured in blood serum (Fig 1B and 1E). We selected IgA from the blood because it provides weekly host information, compared to IgA from the gastrointestinal mucus, which shows similar trends but was available only at sacrificing time. The species-specific IgA response was estimated using ELISA and adult worms as a source of antigen in single infections, while we used excretory-secretory (ES) products from adult parasites for rabbits with dual infections. The choice to use ES products was necessary to minimize cross-reactivity in the antibody response [32]. Specific IgA was provided as Optical Density (O.D.) index while IL4, quantified using qRT-PCR, was available as mRNA gene expression $2^{-\Delta \Delta C_{t}}$ where the gene expression value, *C*_*t*_, from infected rabbits was scaled over the housekeeping gene, HPRT, and control animals. Parasite intensities were estimated using aliquots and standard parasitological techniques. The complete description of experimental design, sample collection and laboratory analyses is reported in Murphy et al. [31, 32].

The selection of IL4 and specific IgA was based on previous studies on this system and general findings from helminth immunology. Specifically, the Boolean modeling of the complete network of compartmentalized immune responses to *T. retortaeformis* suggested that IL4 and species-specific IgA play an important role in the reduction, and possible removal, of this parasite [33]. A relationship between parasite intensity and specific IgA, and/or IL4, was also found using Principal Component Analysis and rabbits with single and dual infections [31, 32]. Similar immune interactions were also found for *G. strigosum*, although there was no evidence of parasite clearance [31, 32]. Recent laboratory trials showed that specific IgA was negatively associated with *T. retortaeformis* intensity and body length in dual infected rabbits, and *G. strigosum* body length in single infected rabbits [34]. More broadly, IL4 and IgA have been identified to be important components in the anti-inflammatory type 2 reaction against gastrointestinal helminths [29, 30, 35].

### Single infection: Model description, selection and calibration

Our within-host model of single infection explicitly tracks changes in parasite intensity, *P*, species-specific IgA optical density index level, *I*_1_, and degree of IL4 gene expression *I*_2_ (this latter modeled as a precursor of *I*_1_) at time *t* of the infection, for each helminth independently (*i* = TR for *T. retortaeformis* or GS for *G. strigosum*) as:

$$\begin{array}{c} \left\{ \begin{array}{cc} dP_{i}/dt & =\sigma_{i}L_{0i}e^{\left(-k_{i}t\right)}-\mu P_{i}-\alpha_{i}I_{1i}P_{i}\\ dI_{1i}/dt & =\beta_{1i}{I_{1i}}^{a_{i}}{I_{2i}}^{c_{i}}{P_{i}}^{d_{i}}-\delta_{1i}I_{1i}+\Lambda_{1i}\\ dI_{2i}/dt & =\beta_{2i}{I_{2i}}^{b_{i}}P_{i}-\delta_{2i}I_{2i}+\Lambda_{2i} \end{array}\right. \end{array}$$ (1)

where *L*_0*i*_ is the initial larval inoculum, *σ*_*i*_ is the rate at which larvae survive to the adult stage, *k*_*i*_ is the development rate of larvae into adults (1/*k*_*i*_ being the average development time), *μ* is the baseline mortality rate of the established adults and *α*_*i*_ is the mortality/expulsion rate caused by the specific IgA response, *I*_1_, to the parasite *i*. In the absence of parasites (*P*_*i*_ = 0), *I*_1*i*_ is mantained at the equilibrium concentration $\bar{I_{1i}}=\Lambda_{1i}/\delta_{1i}$, which is given by the ratio between the baseline production rate Λ_1*i*_ and the decay rate *δ*_1*i*_. Likewise, the IL4 response, *I*_2*i*_, has a baseline production rate Λ_2*i*_ and a decay rate *δ*_2*i*_ that, in absence of the parasite (*P*_*i*_ = 0), leads to an equilibrium concentration $\bar{I_{2i}}=\Lambda_{2i}/\delta_{2i}$. Conversely, in the presence of infection, the production of *I*_1*i*_ and *I*_2*i*_ is assumed to be stimulated by the immune response through the activation coefficients *β*_1*i*_ and *β*_2*i*_. Table 1 reports the complete parameter details.

**Table 1:** Single infection: Model parameters, definitions, dimensions and available values for *T. retortaeformis* (TR) and *G. strigosum* (GS).

Parameter	Definition	Unit	TR-Value	GS-Value
*σ*	Larvae survival rate	d^-1^	to be calibrated	to be calibrated
*L*_0_	Initial larval inoculum	number of L_3_ larvae	5500	650
*k*	Larvae development rate	d^-1^	1/*k* = 4 d	1/*k* = 14 d
*μ*	Natural parasite mortality rate	d^-1^	2.74 × 10^-3^	
*α*	IgA-induced parasite mortality rate	d^-1^ O.D.Index^-1^	to be calibrated	to be calibrated
*β*_1_	Coefficient of IgA activation	d^-1^ mRNA^-1^ IOI^-1^	to be calibrated	to be calibrated
${\bar{I}}_{1}$	IgA equilibrium value	O.D.Index	9.05 × 10^-2^	0.875
*δ*_1_	IgA natural decay rate	d^-1^	4.97 × 10^-2^	2.55 × 10^-2^
Λ_1_	IgA baseline production rate	O.D.Index d^-1^	4.50 × 10^-3^	2.23 × 10^-2^
*a*	Exponent of autocatalytic IgA production	-	to be calibrated	to be calibrated
*c*	Exponent of IgA production stimulated by IL4	-	to be calibrated	to be calibrated
*d*	Exponent of IgA production stimulated by P	-	0 or 1	0 or 1
*β*_2_	Coefficient of IL4 activation	d^-1^ IOI^-1^	to be calibrated	to be calibrated
${\bar{I}}_{2}$	IL4 equilibrium value	mRNA	1	1
*δ*_2_	IL4 natural decay rate	d^-1^	1.37 × 10^-2^	7.44 × 10^-2^
Λ_2_	IL4 baseline production rate	mRNA d^-1^	1.37 × 10^-2^	7.44 × 10^-2^
*b*	Exponent of autocatalytic IL4 production	-	to be calibrated	to be calibrated

To examine how IgA and IL4 interact and affect parasite intensity, and viceversa, we assume that *I*_2_ is linearly activated by the parasite *P*. Then, four main hypotheses are investigated:

1. *I*_1_ production is autocatalytic with a power law of coefficient *a*_*i*_;
2. *I*_2_ production is autocatalytic with a power law of coefficient *b*_*i*_;
3. *I*_1_ is activated by IL4 with a power law of exponent *c*_*i*_;
4. *I*_1_ is independent from parasite intensity (*d*_*i*_ = 0) otherwise linearly activated by the parasite through the coefficient (*d*_*i*_ = 1).

These hypotheses generate 16 competing models that selectively evaluate different mechanisms of host-parasite interaction and responses (Table 2).

**Table 2:** Tested hypotheses and related mechanisms for the competing models of single infection. The parameters *a*, *b* and *c* are set equal to 0 when the respective mechanism is not considered, otherwise they are calibrated (To be cal.).

Model	Hypotheses/Mechanisms	*a*	*b*	*c*	*d*
M1	Null model (no autocatalysis and no interaction between IgA and IL4)	0	0	0	0
M2	IgA autocatalytic	To be cal.	0	0	0
M3	IL4 autocatalytic	0	To be cal.	0	0
M4	IgA stimulation by IL4	0	0	To be cal.	0
M5	IgA autocatalytic + IL4 autocatalytic	To be cal.	To be cal.	0	0
M6	IgA autocatalytic + IgA stimulation by IL4	To be cal.	0	To be cal.	0
M7	IL4 autocatalytic + IgA stimulation by IL4	0	To be cal.	To be cal.	0
M8	IgA autocatalytic + IL4 autocatalytic + IgA stimulation by IL4	To be cal.	To be cal.	To be cal.	0
M9	Null model (no interaction between IgA and IL4) + IgA stimulation by P	0	0	0	1
M10	IgA autocatalytic + IgA stimulation by P	To be cal.	0	0	1
M11	IL4 autocatalytic + IgA stimulation by P	0	To be cal.	0	1
M12	IgA stimulation by IL4 + IgA stimulation by P	0	0	To be cal.	1
M13	IgA autocatalytic + IL4 autocatalytic + IgA stimulation by P	To be cal.	To be cal.	0	1
M14	IgA autocatalytic + IgA stimulation by IL4 + IgA stimulation by P	To be cal.	0	To be cal.	1
M15	IL4 autocatalytic + IgA stimulation by IL4 and P	0	To be cal.	To be cal.	1
M16	IgA autocatalytic + IL4 autocatalytic + IgA stimulation by IL4 and P	To be cal.	To be cal.	To be cal.	1

The baseline equilibrium value of IgA in the blood, $\bar{I_{1i}}$ and IL4, $\bar{I_{2i}}$, at the site of the infection are available from the control rabbits [31, 32]. The decay rates of specific IgA, *δ*_1*i*_, and IL4, *δ*_2*i*_, are provided by infections on the same system, where rabbits were treated with an anthelmintic and the immune response quantified just before the treatment and one month later, during which animals were kept untouched [34]. The baseline production rates Λ_1*i*_ and Λ_2*i*_ is estimated as $\bar{I_{1i}}\delta_{1i}$ and $\bar{I_{2i}}\delta_{2i}$, from Murphy et al. [31]. The development rate of larvae into adults, *k*_*i*_, is assumed to be fixed but different between the two helminths [36, 37], while natural parasite mortality rate, *μ*, is assumed to be mainly caused by the natural mortality rate of the host, whose lifespan has been set equal to one year [38]. The remaining parameters *σ*_*i*_, *α*_*i*_, *β*_1*i*_, *β*_2*i*_, *a*_*i*_, *b*_*i*_ and *c*_*i*_ are calibrated to estimate the contribution and degree of responses of *P*, *I*_1_ and *I*_2_.

Model calibration was performed by minimizing the following error function, *ERR*, calculated as a weighted sum of the variable under study errors, using their sample size as weights, *n*_*P*_, $n_{I_{1}}$ and $n_{I_{2}}$:

$$\begin{array}{c} ERR=n_{P}ERR_{P}+n_{I_{1}}ERR_{I_{1}}+n_{I_{2}}ERR_{I_{2}} \end{array}$$ (2)

Since the variables are characterized by a different magnitude and sample size, we consider the percentage errors to compare them. Each error component of Eq 2 is thus computed as the logarithmic square ratio between observed and estimated values, normalized by data sample size:

$$\begin{array}{c} ERR_{P}=log(\frac{\sum_{j=1}^{n_{P}}{\left[\frac{{\tilde{P}}_{j}}{\hat{P_{j}}}\right]}^{2}}{n_{P}}),\;ERR_{I_{1}}=log(\frac{\sum_{J=1}^{n_{I_{1}}}{\left[\frac{{\tilde{I}}_{1J}}{{\hat{I}}_{1J}}\right]}^{2}}{n_{I_{1}}}),\;ERR_{I_{2}}=log(\frac{\sum_{j=1}^{n_{I_{2}}}{\left[\frac{{\tilde{I}}_{2j}}{{\hat{I}}_{2j}}\right]}^{2}}{n_{I_{2}}}) \end{array}$$ (3)

Here, ${\tilde{P}}_{j}$ and ${\tilde{I}}_{2j}$ represent the observed parasite intensity and IL4 response, respectively, for each rabbit *j*, while ${\tilde{I}}_{1J}$ is the species-specific IgA response from serum sample *J*; ${\hat{P}}_{j}$, ${\hat{I}}_{2j}$ and ${\hat{I}}_{1J}$ are the estimated values of the considered variables. Sampling times, $\tilde{t}$, differ for the three variables: it represents the fixed time points when the cross-sectional data *P* and *I*_2_ are collected from four sacrificed rabbits, while it identifies the longitudinal time sampling of *I*_1_ from the blood of every rabbit still alive at time $\tilde{t}$. We select the best model, among the candidate set for each helminth, based on the best compromise between goodness of fit and parsimony, according to the Akaike Information Criterion (*AIC*). Specifically, for each model we evaluated the score *AIC* = *ERR* + 2*h*, where *ERR* represents the minimized error function (see Eq 2) and *h* is the model complexity, i.e. the number of parameters to calibrate [39, 40].

### Dual infection: Model description, selection and calibration

For rabbits infected with both helminths, we coupled the single infection models by considering different scenarios of immune mediated interaction between *T. retortaeformis* and *G. strigosum*. Given that the two helminths inhabit different organs, and based on previous work on this rabbit-helminth system [32, 34], we did not address possible ecological interference via parasite excretory/secretory products or indirect competition for resources. New immune pathways and variables can be activated in the presence of different helminth species, here, we explore how IL4 and specific IgA could be stimulated in dual infections.

We assumed that helminth interactions occur at the level of antibodies, namely, by affecting their production, a process here identified as *cross-immune activation*, and/or their ability to clear the co-infecting parasite, here indicated as *cross-immunity*. By cross-immune activation we refer to the stimulation of specific IgA via a power law function by IL4 being produced against the second parasite species. By cross-immunity we indicate specific IgA that, stimulated by its own IL4 and directed against its specific helminth, can also target the second parasite (Table 3).

**Table 3:** Dual infection: Model parameters, definitions, dimensions and available values for both *T. retortaeformis* (TR) and *G. strigosum* (GS); model parameters that are not reported in the table are assumed to be equal to single-infection values (see Table 1).

Parameter	Definition	Unit	Value [CI]
*α*_*TR*_	TR specific IgA that induces TR mortality rate	d^-1^O.D.Index^-1^	to be calibrated
*α*_*GS*_	GS specific IgA that induces GS mortality rate	d^-1^O.D.Index^-1^	to be calibrated
*α*_*GSonTR*_	GS specific IgA that induces TR mortality rate	d^-1^ O.D.Index^-1^	to be calibrated
*α*_*TRonGS*_	TR specific IgA that induces GS mortality rate	d^-1^ O.D.Index^-1^	to be calibrated
*β*_*GSonTR*_	Coefficient of TR-specific IgA activation stimulated by GS	d^-1^ mRNA^-1^ IOI^-1^	to be calibrated
*β*_*TRonGS*_	Coefficient of GS-specific IgA activation stimulated by TR	d^-1^ mRNA^-1^ IOI^-1^	to be calibrated
${\bar{I}}_{1TR}$	TR IgA equilibrium value	d^-1^	0.248
*δ*_1*TR*_	TR IgA natural decay rate	d^-1^	1.33 × 10^-2^
Λ_1*TR*_	TR IgA baseline production rate	O.D.Index d^-1^	2.80 × 10^-3^
${\bar{I}}_{2TR}$	TR IL4 equilibrium value	mRNA	1
*δ*_2*TR*_	TR IL4 natural decay rate	d^-1^	1.55 × 10^-2^
Λ_2*TR*_	TR IL4 baseline production rate	mRNA d^-1^	1.55 × 10^-2^
${\bar{I}}_{1GS}$	GS IgA equilibrium value	O.D.Index	0.376
*δ*_1*GS*_	GS IgA natural decay rate	d^-1^	2.07 × 10^-2^
Λ_1*GS*_	GS IgA baseline production rate	O.D.Index d^-1^	7.77 × 10^-3^
${\bar{I}}_{2GS}$	GS IL4 equilibrium value	mRNA	1
*δ*_2*GS*_	GS IL4 natural decay rate	d^-1^	7.25 × 10^-2^
Λ_2*GS*_	GS IL4 baseline production rate	mRNA d^-1^	7.25 × 10^-2^

The full version of the dual-infection model that accounts for all the helminth interactions and immune processes (Tables 2 and 4) is the following:

$$\begin{array}{c} \left\{ \begin{array}{cc} dP_{TR}/dt & =\sigma_{TR}L_{0TR}e^{\left(-k_{TR}t\right)}-\mu P_{TR}-\alpha_{TR}I_{1TR}P_{TR}-\alpha_{GSonTR}I_{1GS}P_{TR}\\ dP_{GS}/dt & =\sigma_{GS}L_{0GS}e^{\left(-k_{GS}t\right)}-\mu P_{GS}-\alpha_{GS}I_{1GS}P_{GS}-\alpha_{TRonGS}I_{1TR}P_{GS}\\ dI_{1TR}/dt & =\beta_{1TR}{I_{1TR}}^{a_{TR}}{I_{2TR}}^{c_{TR}}{P_{TR}}^{d_{TR}}+\beta_{1GSonTR}{I_{2GS}}^{c_{GSonTR}}-\delta_{1TR}I_{1TR}+\Lambda_{1TR}\\ dI_{1GS}/dt & =\beta_{1GS}{I_{1GS}}^{a_{GS}}{I_{2GS}}^{c_{GS}}{P_{GS}}^{d_{GS}}+\beta_{1TRonGS}{I_{2TR}}^{c_{TRonGS}}-\delta_{1GS}I_{1GS}+\Lambda_{1GS}\\ dI_{2TR}/dt & =\beta_{2TR}{I_{2TR}}^{b_{TR}}P_{TR}-\delta_{2TR}I_{2TR}+\Lambda_{2TR}\\ dI_{2GS}/dt & =\beta_{2GS}{I_{2GS}}^{b_{GS}}P_{GS}-\delta_{2GS}I_{2GS}+\Lambda_{2GS} \end{array}\right. \end{array}$$ (4)

**Table 4:** Tested hypotheses and related mechanisms for the competing models of helminths interaction in dual infection. The parameters *α*_*GSonTR*_, *α*_*TRonGS*_, *β*_1*GSonTR*_ and *β*_1*TRonGS*_ are set equal to 0 when the respective mechanism is not considered, otherwise they are calibrated (To be cal.). Further hypotheses tested are listed in Table 2.

Model	Hypotheses/mechanisms	*α*_*GSonTR*_, *α*_*TRonGS*_	*β*_1*GSonTR*_, *β*_1*TRonGS*_
M1	No parasite interaction	0	0
M2	IgA-cross immunity	To be cal.	0
M3	IgA cross-immune activation	0	To be cal.
M4	IgA cross-immunity + IgA cross-immune activation	To be cal.	To be cal.

In addition to the components already described for the single infection model (Table 1), here: *α*_*GSonTR*_ represents the cross-immunity of the specific IgA response stimulated by and produced against *G. strigosum* that also attacks *T. retortaeformis*, vice versa *α*_*TRonGS*_ is the response to *T. retortaeformis* that also attacks *G. strigosum*; $\beta_{1GSonTR}{I_{2TR}}^{c_{GSonTR}}$ and $\beta_{1TRonGS}{I_{2GS}}^{c_{TRonGS}}$ are, respectively, the immune activation of IgA specific to *T. retortaeformis* by IL4 to *G. strigosum*, and the same for IgA specific to *G. strigosum* being activated by IL4 to *T. retortaeformis*. To reduce model complexity while retaining the fundamental mechanisms of regulation, we assumed that the parameters *k*_*TR*_, *k*_*GS*_, *μ*, *σ*_*TR*_, *σ*_*GS*_, *β*_1*TR*_, *β*_1*GS*_, *β*_2*TR*_, *β*_2*GS*_ are taken from the single-infection best-selected model. This assumption was also applied to the exponents *a*_*TR*_, *a*_*GS*_, *b*_*TR*_, *b*_*GS*_, *c*_*TR*_, *c*_*GS*_ and *d*_*TR*_, *d*_*GS*_. To improve model identifiability in the calibration phase, we fixed the exponents *c*_*GSonTR*_ and *c*_*TRonGS*_ of the IgA immune activation to the values calibrated in single infection, *c*_*GS*_ and *c*_*TR*_, respectively. Here, we assumed that IgA specific to the first helminth responds with the same power law to IL4, *I*_2_, whether this is stimulated by the first or second helminth, but with a different rate between the two parasites (*β*_1*GS*_ ≠ *β*_1*TRonGS*_ and *β*_1*TR*_ ≠ *β*_1*GSonTR*_). Likewise for single infections, the natural IgA and IL4 decay rates, *δ*_1*TR*_, *δ*_1*GS*_, *δ*_2*TR*_ and *δ*_2*GS*_, and their equilibrium values, ${\bar{I}}_{1TR}$, ${\bar{I}}_{1GS}$, ${\bar{I}}_{2TR}$, ${\bar{I}}_{2GS}$, were available from control animals [32, 34]. Moreover, the baseline productions for specific IgA, Λ_1*TR*_, Λ_1*GS*_, and IL4, Λ_2*TR*_, Λ_2*GS*_, were estimated as in single infections. A complete parameter description of the dual-infection model is reported in Table 3.

For dual infections, we tested different mechanisms of helminth interaction and all their possible combinations. We also included the hypotheses tested in single infections and considered a scenario with no interaction between the two helminths. The complete list of tested models is reported in Tables 2 and 4.

Given the methodological change in the quantification of specific IgA between single and dual infection, IgA-induced parasite mortality parameters *α*_*TR*_ and *α*_*GS*_ were recalibrated for the dual infection [32]. In addition to *α*_*TR*_ and *α*_*GS*_, the parameters *α*_*GSonTR*_, *α*_*TRonGS*_, *β*_1*GSonTR*_, *β*_1*TRonGS*_ were calibrated by minimizing the previously described error function simultaneously for the two parasites,

$$\begin{array}{c} ERR=ERR_{TR}+ERR_{GS} \end{array}$$ (5)

where

$$\begin{array}{c} ERR_{TR}=n_{P_{TR}}ERR_{P_{TR}}+n_{I_{1TR}}ERR_{I_{1TR}}+n_{I_{2TR}}ERR_{I_{2TR}} \end{array}$$ (6)

and

$$\begin{array}{c} ERR_{GS}=n_{P_{GS}}ERR_{P_{GS}}+n_{I_{1GS}}ERR_{I_{1GS}}+n_{I_{2GS}}ERR_{I_{2GS}}. \end{array}$$ (7)

Each error term $ERR_{P_{TR}}$, $ERR_{P_{GS}}$, $ERR_{I_{1TR}}$, $ERR_{I_{1GS}}$, $ERR_{I_{2TR}}$ and $ERR_{I_{2GS}}$ was computed as for single infections (see Eq 3). We evaluated our hypotheses, and the resulting models, using the Akaike Information Criterion as discussed for single infections.

### Empirical probability distributions of estimated parameters

The best models selected for single and dual infections are used to assess the empirical probability distributions of the estimated parameters by means of the bootstrap technique [41]. This approach allows us to take into account parameter uncertainty and evaluate how this translates into model outputs. Briefly, we reconstructed 1000 replicates of bootstrapped time series by randomly sampling with replacement the three observed variables: parasite intensity, *P*, species-specific IgA, *I*_1_, and IL4, *I*_2_. For every single infection model we assessed the parameter values (*σ*_*i*_, *α*_*i*_, *β*_1*i*_, *β*_2*i*_, *a*, *b* and *c*) for each replicate series by minimizing the error function (Eq 2). For the dual infection model we considered the previously obtained probability distributions of parameters that are fixed to single infection values (*σ*_*TR*_, *σ*_*GS*_, *β*_1*TR*_, *β*_1*GS*_, *β*_2*TR*_, *β*_2*GS*_, *a*_*TR*_, *a*_*GS*_, *b*_*TR*_, *b*_*GS*_, *c*_*TR*_, and *c*_*GS*_), and calibrated the remaining parameters, (*α*_*TR*_, *α*_*GS*_, *α*_*GSonTR*_, *α*_*TRonGS*_, *β*_1*GSonTR*_ and *β*_1*TRonGS*_), by obtaining their distributions, and by minimizing the dual infection error function (Eq 7).

## Results

### Empirical laboratory observations

Following the initial larval inoculum, *T. retortaeformis* intensities start declining at around day 15^th^ in both single and dual infections (Fig 1A), while *G. strigosum* remains high throughout the experiments (Fig 1D). Specific IgA to *T. retortaeformis* quickly builds in the first 20 days post infection and declines thereafter for single but not dual infections (Fig 1B). Specific IgA to *G. strigosum* remains consistently high in the two types of infection (Fig 1E). IL4 against *T. retortaeformis* single infection shows a tendency to peak around 30 days post infection while an earlier peak at day 15^th^ is observed in the dual infection (Fig 1C). IL4 against *G. strigosum* peaks around 45 days post inoculum in both single and dual infected animals, and shows higher variation in the latter group (Fig 1F). A comparison of parasite intensity and IL4 between single and dual infected hosts, using Generalized Linear Models (GLM), shows a significant difference in IL4 expression for *T. retortaeformis*; no other significant relationships were found (S1 Table). Specific-IgA was not compared because of the different antigen measurements (See Materials and methods).

### Simulations from models of single infection

**Fig 2:**
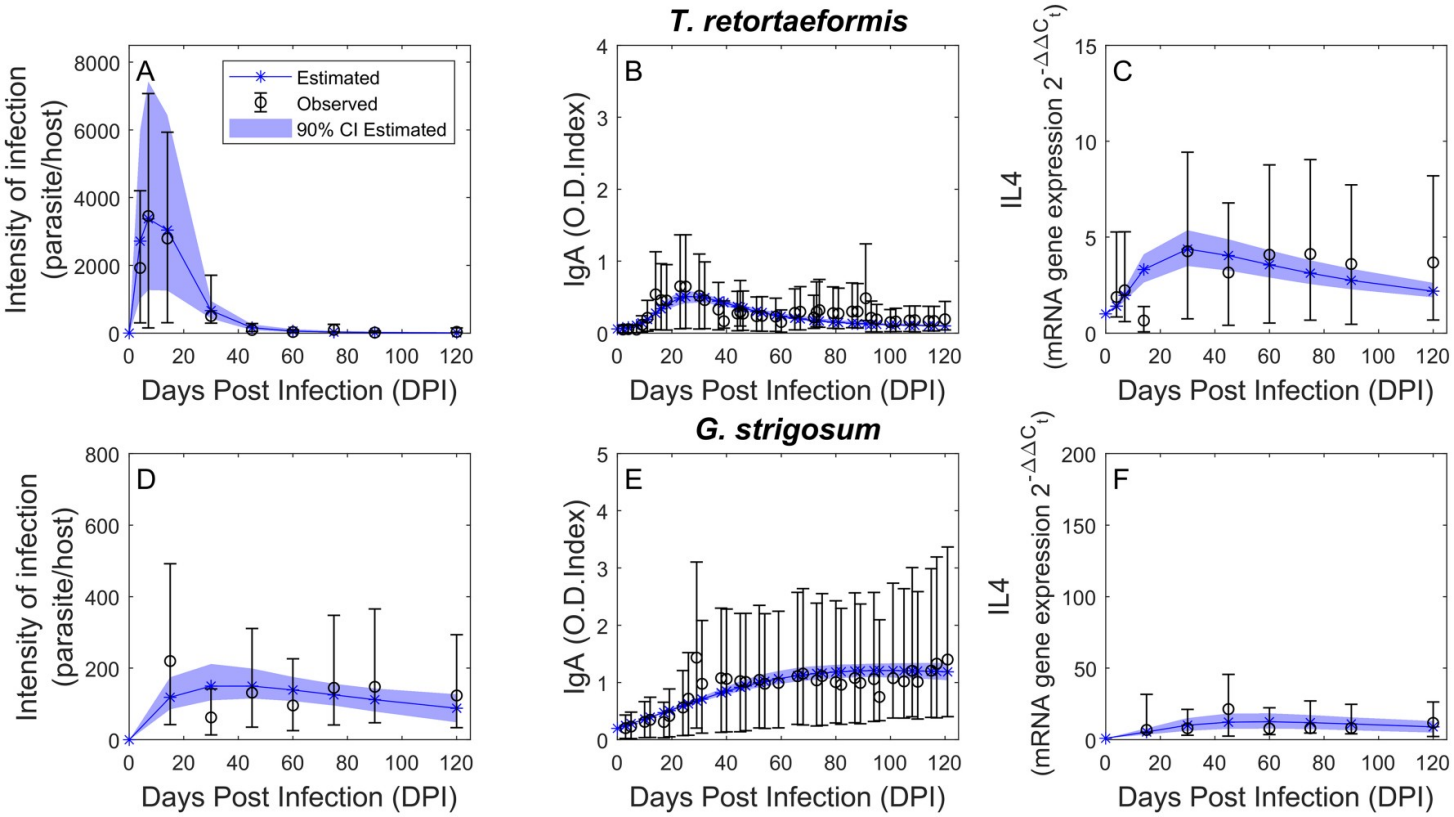
Single infection simulations (blue) and observation data (black). Mean intensity of infection (A and D), specific IgA response estimated using adult worms as a source of antigen (B and E), and IL4 expression (C and F) over the course of the infection. Observed data (geometric mean multiplied/divided by the S.D. error factor (circle)) and estimated values (star) with the relative 90% confidence interval (shade) are reported for both *T. retortaeformis* (A-C) and *G. strigosum* (D-F).

Among the 16 hypothesis-driven models tested, the option that best captures the dynamics of infection for both helminths is M12, which includes IgA stimulation by IL4 and by the parasite, *P*, and excludes the other mechanisms (Tables 5 and 6). For both parasites, the least performing models lack the interaction between parasite abundance and IgA production. The worst model for *T. retortaeformis* assumes that IgA production is both autocatalytic and stimulated by IL4, while for *G. strigosum* is based on the assumption that IL4 production is an autocatalytic process. The role of IL4 on IgA activation and amplification, and the relative feedback on IL4, is quite complex [42]. We simplified this relationship by testing a direct and positive effect of IL4 on IgA production. The estimated value of the shape parameter *c* (Table 6) shows that the effect of IL4 on IgA is stronger for *T. retortaeformis* than *G. strigosum* (*c*_*TR*_ > *c*_*GS*_), while the stimulus to specific IgA production is lower for the earlier than the latter (*β*_1*GS*_ > *β*_1*TR*_). The impact of IgA on helminth mortality is higher for *T. retortaeformis* than *G. strigosum* (*α*_*TR*_ > *α*_*GS*_) and contributes to the fast removal of the former. Simulations show that, following the initial establishment in the small intestine, the estimated *T. retortaeformis* intensity rapidly decreases from 15 days post infection and remains low for the rest of the trial (Fig 2A). The estimated specific IgA follows a similar trend with a peak at around 3 weeks post infection and a decrease thereafter (Fig 2B). The estimated IL4 is also consistent with the empirical data (Fig 2C).

**Table 5:** Summary of competing models for single infections based on performance and level of complexity. Model complexity, *h*, the contribution of each variable to the error function (*ERR*_*P*_, $ERR_{I_{1}}$ and $ERR_{I_{2}}$; see Eq 3), *AIC* and Δ*AIC* are reported for both *T. retortaeformis* and *G. strigosum*.

		*T. retortaeformis*					*G. strigosum*				
Model	*h*	*ERR*_*P*_	$ERR_{I_{1}}$	$ERR_{I_{2}}$	*AIC*	Δ*AIC*	*ERR*_*P*_	$ERR_{I_{1}}$	$ERR_{I_{2}}$	*AIC*	Δ*AIC*
M1	4	3.262	0.9198	0.4374	-27.61	113.1	0.4714	0.5482	1.687	-284.0	15.72
M2	5	3.262	0.9198	0.4374	-25.61	115.1	0.4714	0.5384	1.687	-290.6	9.144
M3	5	3.220	0.9198	0.3839	-31.36	109.3	0.4714	0.5482	1.687	-281.98	17.72
M4	5	3.262	0.9198	0.4374	-25.61	115.1	0.4714	0.5321	1.688	-296.1	3.588
M5	6	3.220	0.9198	0.3839	-29.36	111.3	0.4715	0.5384	1.686	-288.6	11.14
M6	6	3.262	0.9198	0.4374	-23.61	117.1	0.4848	0.5308	1.677	-294.7	5.020
M7	6	3.220	0.9198	0.3839	-29.36	111.3	0.4848	0.5308	1.677	-294.7	5.020
M8	7	3.220	0.9198	0.3839	-27.36	113.3	0.4848	0.5308	1.677	-292.7	7.020
M9	4	3.551	0.7973	0.4176	-111.3	29.43	0.5428	8.610	1.691	-283.98	15.72
M10	5	2.417	0.7810	0.5003	-129.7	10.97	0.4784	0.5308	1.678	-297.0	2.703
M11	5	3.454	0.7985	0.3812	-113.1	27.59	0.5428	8.610	1.691	-282.0	17.72
M12	5	2.449	0.7627	0.5345	-140.7	0	0.4974	0.5266	1.675	-299.7	0
M13	6	2.387	0.7803	0.3799	-139.8	0.9419	0.4784	0.5308	1.678	-295.0	4.703
M14	6	2.449	0.7627	0.5345	-138.7	2	0.4974	0.5266	1.675	-297.7	2
M15	6	2.449	0.7627	0.5345	-138.7	2	0.4974	0.5266	1.675	-297.7	2
M16	7	2.387	0.7803	0.3799	-137.8	2.942	0.4974	0.5266	1.675	-295.7	4

**Table 6:** Single infections: Estimated values and 90% confidence intervals (CI) for the parameters of the selected model (M12) for *T. retortaeformis* (TR) and *G. strigosum* (GS). The 90% CIs are estimated via bootstrap.

Parameter	TR-Value	TR-[CI]	GS-Value	GS-[CI]
*σ*	0.203	[0.075;0.45]	2.04 × 10^-2^	[1.46;3.09] × 10^-2^
*α*	0.215	[0.141;0.277]	4.21 × 10^-3^	[0;12.6] × 10^-3^
*β*_1_	1.20 × 10^-6^	[0.215;2.07] × 10^-6^	1.10 × 10^-5^	[0.054;4.61] × 10^-5^
*c*	2.00	[1.07;2.90]	0.870	[0.252;2.09]
*β*_2_	6.4 × 10^-5^	[3.44;13.4] × 10^-5^	5.77 × 10^-3^	[3.74;8.27] × 10^-3^

For *G. strigosum* simulations indicated a weak IL4 signal to IgA production, namely a low *c*, and a low parasite mortality induced by specific IgA, *α*_*GS*_ (Fig 2D). Simulated time series are consistent with the average empirical trends (Fig 2D, 2E and 2F). Specifically, mean intensity captures well the dynamics of infection (Fig 2D), specific IgA follows tightly the laboratory data by slowly increasing to an asymptote at around 50 days post infection (Fig 2E), while mean IL4 peaks at around 45-60 days post infection (Fig 2F).

Overall, we show that the activated immunological pathway is the same for both helminths, precisely, specific IgA is stimulated by IL4 and by the intensity of the target parasite. However, differences in the extent of these relationships and signals lead to the contrasting dynamics of infection observed.

We retrieved the empirical probability distributions of the estimated parameters and evaluated how parameter uncertainty translated into model outputs. We calculated the 90% CI for each model variable, running the model for all the estimated quintets of parameter values. The distributions of *α* and *c* for *T. retortaeformis* are symmetric and centered around the calibrated values, while those of *β*_1_, *β*_2_ and *σ* are right skewed distributed (S1 Fig). For *G. strigosum*, the empirical probability distributions are all left skewed (S2 Fig). Some of the resulting correlations between parameters are in accordance with their biological contribution and role in the model, for both helminths. Specifically, parasites regulation by specific IgA becomes stronger and more selective (i.e. highly effective) when the stimulus to produce antibodies and/or IL4 decreases (*α* is negatively correlated with *β*_1_ and *β*_2_), a trend apparent for *T. retortaeformis*. Lower regulation also facilitates larval survival (*α* is positively correlated with *σ*) in both helminths. Similarly, the specific IgA stimulation by IL4 is greater if IgA or IL4 production decreases (*c* is negatively correlated with *β*_1_ and *β*_2_).

### Simulations from models of dual infection

For rabbits with both helminths, the selected model M3 indicates that parasite dynamics are driven by multiple immune activation pathways, specifically, the regulation by their specific IL4-IgA response, as described in single infections, and the additional effect from the activation of their specific IgA via IL4 produced against the second helminth (Tables 7 and 8). The second best-performing model, M4, includes both IgA immune-activation and IgA cross-immunity, however, framework complexity is higher and overall performance is lower (Δ*AIC* ≫2). The performance of the remaining models is consistently lower.

**Table 7:** Summary of competing models for the dual infection based on performance and level of complexity. Model complexity, *h*, the contribution of each variable to the error function (*ERR*_*P*_, $ERR_{I_{1}}$ and $ERR_{I_{2}}$; see Eq 3), *AIC* and Δ*AIC* are reported.

Model	*h*	$ERR_{P_{TR}}$	$ERR_{I_{1}TR}$	$ERR_{I_{2}TR}$	$ERR_{P_{GS}}$	$ERR_{I_{1}GS}$	$ERR_{I_{2}GS}$	*AIC*	Δ*AIC*
M1	2	5.929	0.3591	0.3965	0.3610	0.2005	1.926	-579.5	35.55
M2	4	5.929	0.3591	0.3965	0.3610	0.2005	1.926	-575.5	39.55
M3	4	7.027	0.3022	0.3997	0.3609	0.1958	1.926	-615.0	0
M4	6	7.027	0.3022	0.3997	0.3609	0.1958	1.926	-611.0	4

**Table 8:** Dual infection: Estimated values and 90% confidence intervals (CI) of the selected model (M3) parameters. The 90% CIs are estimated via bootstrap.

Parameter	Value	[CI]
*α*_*TR*_	0.182	[0.1152;0.2401]
*α*_*GS*_	2.959 × 10^-22^	[1.566 × 10^-23^;2.502 × 10^-14^]
*β*_*GSonTR*_	5.809 × 10^-5^	[3.774 × 10^-6^;5.041 × 10^-4^]
*β*_*TRonGS*_	7.674 × 10^-4^	[4.358 × 10^-13^;3.566 × 10^-3^]

Parameter estimation (Table 8) suggests that the stimulation of specific IgA caused by the second helminth is stronger against *G. strigosum* (*β*_*TRonGS*_ = 7.67 × 10^-4^) than *T. retortaeformis* (*β*_*GSonTR*_ = 5.81 × 10^-5^). However, parasite mortality induced by specific IgA, stimulated by and developed against its own helminths, is higher for *T. retortaeformis* (*α*_*TR*_ = 0.182) than *G. strigosum* (*α*_*GS*_ = 2.96 × 10^-22^), which is fundamentally null. In other words, *T. retortaeformis* appears to be mainly regulated by an immune reaction stimulated by and developed against this helminth and, secondly, stimulated by the presence of *G. strigosum*. In contrast, *G. strigosum* is exposed to an immune response mainly stimulated by the presence of *T. retortaeformis*, although this reaction is weak and does not prompt an effective control of the former helminth. The net outcome of these interactions leads to a rapid removal of *T. retortaeformis* but no clearance of *G. strigosum*, consistent with patterns reported for single infections.

**Fig 3:**
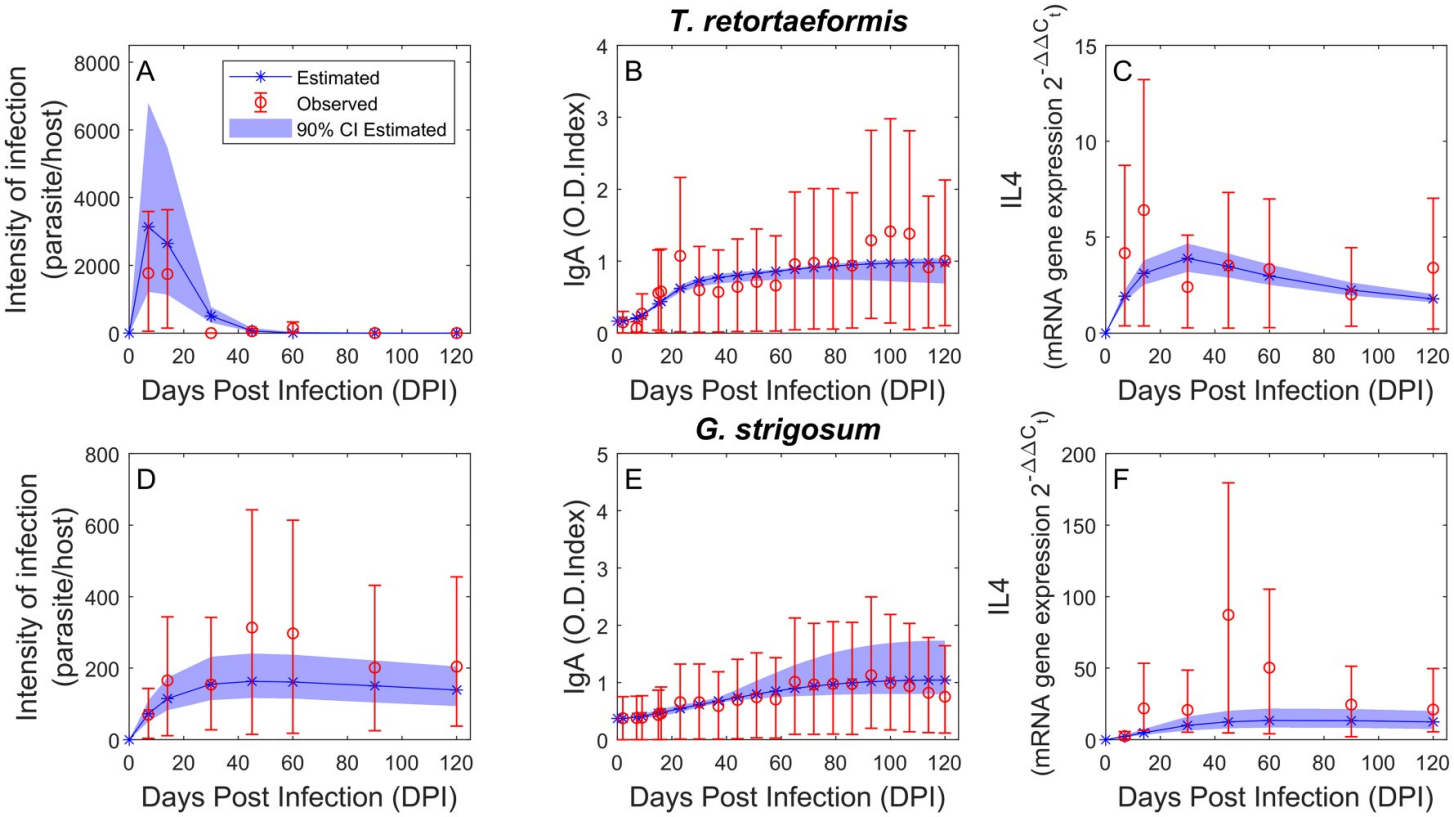
Dual infection simulations (blue) and observation data (red). Mean intensity of infection (A and D), specific IgA response estimated using excretory-secretory (ES) products from adult parasites as a source of antigen (B and E), and IL4 expression (C and F) over the course of the infection. Observed data (geometric mean multiplied/divided by S.D. error factor) and estimated values (star) with the relative 90% CI (shade) are reported for *T. retortaeformis* (A-C) and *G. strigosum* (D-F).

Model simulations well describe the average trends (Fig 3). For *T. retortaeformis*, the simulated time series of mean parasite intensity, IgA and IL4 are consistent with the laboratory data (Fig 3). However, we do note that the mean peak of infection is overestimated, and this coincides with the underestimation of IL4 mean values in the first 40 days post infection, despite the robustness of the estimations and small CIs (Fig 3A and 3C). Similarly, for *G. strigosum*, simulations capture well the mean trend of infection and immune responses over time although there is a tendency for an underestimation of parasite intensity and IL4 around 45-60 days post infection (Fig 3D and 3F).

The empirical probability distributions provide additional details on the role of parameter uncertainty and their relationships to model results. As reported for single infections, we calculated the 90% CIs for each model variable, running the model for all the quartets of calibrated parameters. Distributions of *α*_*GS*_, *β*_*TRonGS*_ and *β*_*GSonTR*_ are left skewed, while *α*_*TR*_ is right skewed (S3 Fig). We found significant negative correlations betweeen *α*_*TR*_ and *β*_*GSonTR*_, confirming the stronger immune regulation of *T. retortaeformis*. A significant but much weaker negative correlation was also found for *G. strigosum*.

Finally, we compare the simulated dynamics between single- and dual-infected rabbits and, although we cannot draw direct analogies for specifc IgA responses, because of the different antigens used, we can highlight some general trends and also examine the intensity of infection and IL4 (S2 Table). Generalized Linear Models (GLM) show that *T. retortaeformis* intensites are significantly lower in dual compared to single infection, while no significant differences are observed for IL4. For *G. strigosum*, dual-infected rabbits have higher IL4 and higher intensities.

Overall, the combination of species-specific and, secondly, cross-stimulated IgA leads to lower intensities and faster clearance of *T. retortaeformis* in dual compared to single infections. In contrast, the remarkably low species-specific and cross-stimulated IgA to *G. strigosum* explains the lack of clearance and the higher intensities in dual-infected rabbits.

## Discussion

We used a modeling selection approach to investigate alternative immunological mechanisms that could explain the within-host dynamics of single and dual infections of two gastrointestinal helminths in rabbits from laboratory trials. By explicitly testing different modes of interaction between key immune variables and parasites we show that the mechanisms of immune regulation are fundamentally conserved between the two helminth species. In other words, the host-parasite interactions are explained by models with the same structural relationships for both helminths. The contrasting dynamics observed are explained by changes in the relative importance of these relationships and the strength of their impact. The control and removal of *T. retortaeformis* is primarily caused by a specific immune response, developed by, and targeting, this parasite. In contrast, the persistence of *G. strigosum* is facilitated by a weak specfic immune reaction, which has no significant impact on parasite mortality/clearance. For rabbits carrying both infections, the selected model includes the activation of new immune pathways, in addition to the mechanisms already identified in single infections. The two helminths interact primarily through an asymmetrical immune stimulation where IL4 produced against the second species stimulates specific IgA to the first helminth. This cross stimulation is weak against both helminths, and has no apparent effect on *G. strigosum*. Overall, the net outcome of specific and cross activated responses leads to the fast removal of *T. retortaeformis* but no significant changes of *G. strigosum*.

In both single and dual infections the selected model indicates that specific IgA is stimulated by IL4 via a power law function. This signal is twice as stronger for *T. retortaeformis* than *G. strigosum* but the activation coefficient for IgA production is lower for the former than the latter. While this would suggest that rabbits are less capable of controlling *T. retortaeformis* than *G. strigosum*, experimental data demonstrate the opposite. We explain this mismatch by showing that helminth mortality, induced by specific IgA, is higher against the first than the second helminth, and this is probably caused by the higher sensitivity of specific IgA to *T. retortaeformis* [43]. Despite the low production of species-specific IgA, the high efficacy against *T. retortaeformis* contributes to its fast removal. In contrast, low production of specific IgA and low mortality of *G. strigosum*, induced by specific IgA, allows the persistence of this helminth in the stomach. The tuning of immune interactions/reactions, where a strong IL4 signal is associated with a low rate of IgA production but a robust ability to attack the helminths, supports the general notion that some immune feedback control is necessary to protect the hosts from eccessive immunopathology, while providing some protection to the parasite burden [34, 42, 44–46].

We tested different modes of helminths interaction. Model selection indicates that the two helminths interact mainly through the activation of specific IgA by IL4 stimulated against the second species. This is a simplification of what is expected to be a more complex immune mechanism, which should involve IL4 and B cells at the systemic level [33]. Our predictions showed that the magnitude of IgA cross-activation is asymmetrical in that it is weaker against *T. retortaeformis* than *G. strigosum*. The alternative model, M4, which includes cross-activation as well as cross-immunity, has a higher level of complexity but lower performance. Therefore, while cross-immunity by specific IgA to ES products should not be dismissed, we presented and discussed the most parsimonious mechanism. Similarly, we cannot dismiss the possibility of a stronger interference between the two helminths through specific IgA against somatic products, however, the observed dynamics of infection suggest that the impact should be relatively low against *G. strigosum* and not significantly strong to cause excessive disturbance against *T. retortaeformis*.

Other mechanisms of interaction could have contributed to the dynamics observed, such as ecological interference via competition for host resources or disturbance through excreted/secreted compounds. The two helminths inhabit different organs and we expect these interactions to have a much weaker impact, expecially for *G. strigosum*. For example, a recent laboratory study that examined changes in parasite abundance and traits before and after drug treatment found no evidence of density-dependent interference between the two helminth intensities [34]. This study suggested that the changes in fecundity observed were explained as driven primarily by processes generated by, and targeting, each species, namely, a specific immune response developed by and directed against *T. retortaeformis* and a weak immune response, with possibly some density-dependent regulation, to *G. strigosum* population. A weak ecological interference was also proposed for rabbits dual infected in the field [43]. In this study, the negative relationship between *G. strigosum* fecundity and intensity of infection in dual infected rabbits was comparable to single infected hosts. For *T. retortaeformis* the fecundity-infection relationship exhibited a posititive trend in dual infected rabbits but a negative pattern in rabbits with single infections, supporting the lack of significant ecological disturbance between the two species.

Our study provides a quantitative explanation of how key immune variables interact, their degree of interaction and the consequences for helminth dynamics in single and dual infections. Type 2 immune pathways have been described in a number of host-helminth systems, and details are available on the contribution of critical constituents [29, 30, 47]. We simplified the immune network by focusing on IL4 and IgA, and we were able to capture their fundamental signals and outcomes during the phases of detection, induction and expulsion of the infection process. IL4 has distinct functions in helminth infections [48] but there is considerable redundancy with IL13, IL5 and IL9, whose contributions also vary among systems and parasite species/strains [30]. In our study simulations indicate that IL4 is critical for parasite regulation through signals that promote IgA production/activation. IgA antibodies have been found to be involved in helminth resistance by affecting abundance and fecundity [17, 49–52], although the degree of response varies among systems and between primary and secondary infections [53–55]. In the present study we show that species-specific IgA well follows the dynamics of both infections and while it affects parasite mortality, particularly for *T. retortaeformis*, it is not sufficiently strong for a sterilizing immunity, consistent with our previous conclusions [33, 56].

The mechanisms of parasite interaction proposed are specific to our rabbit-helmith system and only focus on two important immune variables. Similar processes could be explored for other gastrointestinal helminths where IgA contributes to parasite control, such as *Teladorsagia circumcincta* in sheep [17, 50] or *Strongyloides ratti* in rats [52]. More generally, our model is flexible and can be modified to address specific contexts. For instance, IL4-IgA interactions can be replaced with associations that are more relevant to other systems, for instance, IL4 (or IL5)-IgE (or IgG), or by adding other interactions or variables (e.g. eosinophils or other cytokines), which allows the exploration of alternative mechanisms of the immune network. Similarly, parameters that were kept fixed in single and dual infections can be estimated under different scenarios and degree of responses.

Overall, our framework provides one of the rare examples of within-host dynamics of two closely related helminths, identifies the processes that allow their dynamics and discusses key immune variables that govern these interactions. We show that host heterogeneity in infection and transmission emerges from variations in the strength of the immune relationships. The next critical step is to examine these associations in natural systems by linking within-host processes to parasite dynamics at the host population level through immuno-epidemiological models that explicitly address variation in the immune reactions.

## Supporting information

S1 Fig Empirical probability distributions of model parameters for *T. retortaeformis* (TR) single infection. Parameter (*α*, *β*_1_, *β*_2_, *σ*, *c*) distributions, obtained via 1000 bootstrapped replicates, are reported together with their correlations. In bold, Pearson’s linear correlation coefficient with its significance (* = *p* ≤ 0.05, ** = *p* ≤ 0.01 and *** = *p* ≤ 0.001).
(TIF)

S2 Fig Empirical probability distributions of model parameters for *G. strigosum* (GS) single infection. Parameter (*α*, *β*_1_, *β*_2_, *σ*, *c*) distributions, obtained via 1000 bootstrapped replicates, are reported together with their correlations. In bold, Pearson’s linear correlation coefficient with its significance (* = *p* ≤ 0.05, ** = *p* ≤ 0.01 and *** = *p* ≤ 0.001).
(TIF)

S3 Fig Empirical probability distributions of model parameters from dual infection. Parameter (*α*_*T*_ *R*, *α*_*G*_ *S*, *β*_*TRonGS*_, *β*_*GSonTR*_) distributions, obtained via 1000 bootstrapped replicates, are reported together with their correlations. In bold Pearson’s linear correlation coefficient with its significance (* = *p* ≤ 0.05, ** = *p* ≤ 0.01 and *** = *p* ≤ 0.001).
(TIF)

S1 Table Generalized Linear Model (GLM) comparing the empirical intensity of infection (IOI, assuming a negative binomial distribution with a logarithmic link) or IL4 (assuming a normal distribution) by sampling time (days post-infection, DPI, as continous variable) and single/dual infection (SI/DU, as categorical variable), for *T. retortaeformis* and *G. strigosum*. *AIC* represents the Akaike Information Criterion, while *n* is the sample size.
(PDF)

S2 Table Generalized Linear Model (GLM) comparing the simulated intensity of infection (IOI, assuming a negative binomial distribution with a logarithmic link) or IL4 (assuming a normal distribution) by sampling time (days post-infection, DPI, as continous variable) and single/dual infection (SI/DU, as categorical variable), for *T. retortaeformis* and *G. strigosum*. *AIC* represents the Akaike Information Criterion, while *n* is the sample size.
(PDF)
