# Supplementary figures and images for: Within-host mechanisms of immune regulation explain the contrasting dynamics of two helminth species in both single and dual infections
Source: PLoS Comput Biol. 2020 Nov 23;16(11):e1008438. doi: 10.1371/journal.pcbi.1008438 (PMC7721179; doi:10.1371/journal.pcbi.1008438)

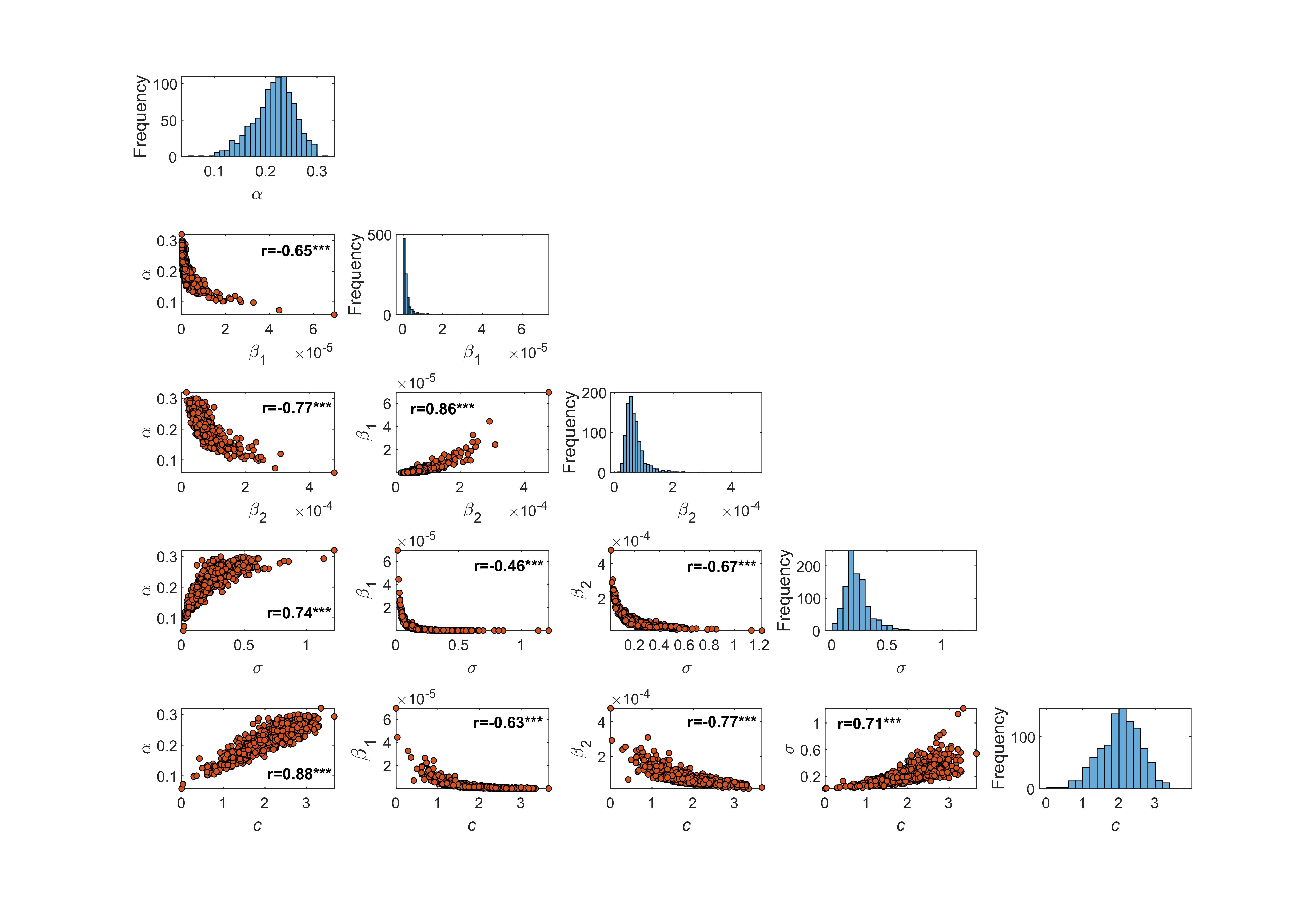

Supplement: S1 Fig — Parameter (α, β1, β2, σ, c) distributions, obtained via 1000 bootstrapped replicates, are reported together with their correlations. In bold, Pearson’s linear correlation coefficient with its significance (* = p ≤ 0.05, ** = p ≤ 0.01 and *** = p ≤ 0.001). (TIF) [file pcbi.1008438.s001.tif]

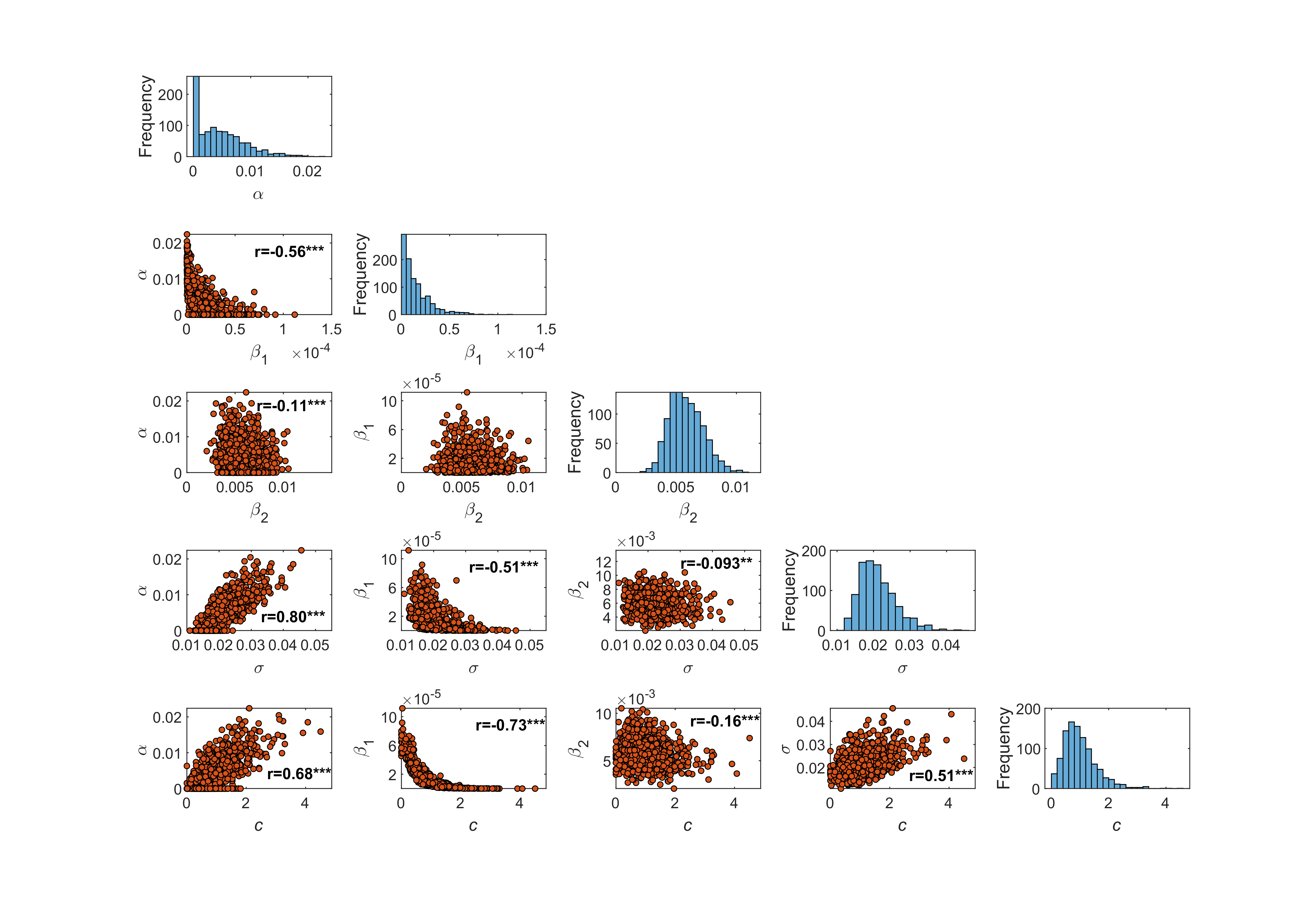

Supplement: S2 Fig — Parameter (α, β1, β2, σ, c) distributions, obtained via 1000 bootstrapped replicates, are reported together with their correlations. In bold, Pearson’s linear correlation coefficient with its significance (* = p ≤ 0.05, ** = p ≤ 0.01 and *** = p ≤ 0.001). (TIF) [file pcbi.1008438.s002.tif]

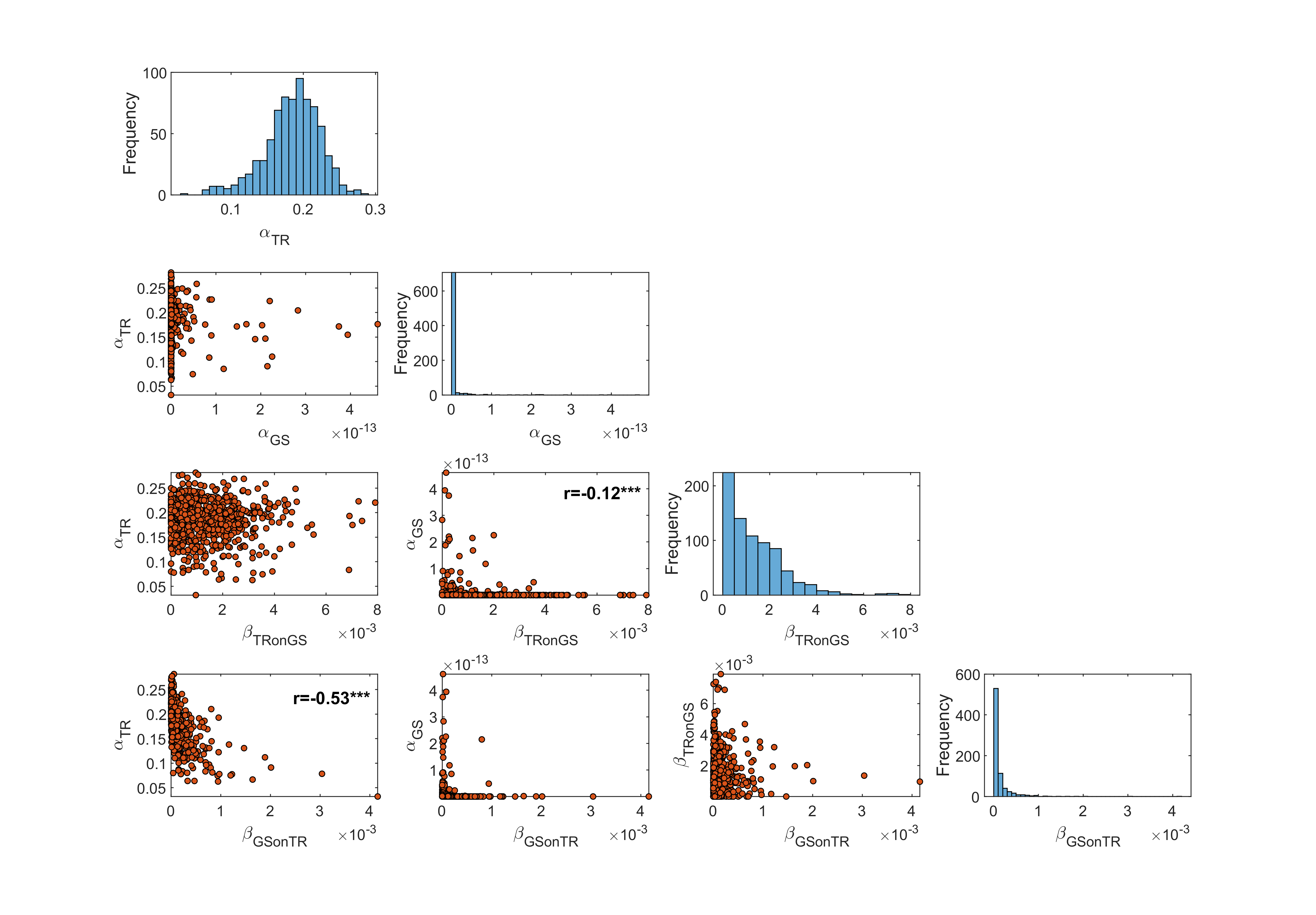

Supplement: S3 Fig — Parameter (αT R, αG S, βTRonGS, βGSonTR) distributions, obtained via 1000 bootstrapped replicates, are reported together with their correlations. In bold Pearson’s linear correlation coefficient with its significance (* = p ≤ 0.05, ** = p ≤ 0.01 and *** = p ≤ 0.001). (TIF) [file pcbi.1008438.s003.tif]
