# Supplementary material for: Within-host mechanisms of immune regulation explain the contrasting dynamics of two helminth species in both single and dual infections
Source: PLoS Comput Biol. 2020 Nov 23;16(11):e1008438. doi: 10.1371/journal.pcbi.1008438 (PMC7721179; doi:10.1371/journal.pcbi.1008438)
Supplement: S1 Table — AIC represents the Akaike Information Criterion, while n is the sample size. (PDF) [file pcbi.1008438.s004.pdf]

S1 Table. Generalized Linear Model (GLM) comparing the observed intensity of infection (IOI, assuming a negative binomial distribution with a logarithmic link) or IL4 (assuming a normal distribution) by sampling time (days post-infection, DPI, as continous variable) and single/dual infection (SI/DU, as categorical variable), for *T. retortaeformis* and *G. strigosum*. *AIC* represents the Akaike Information Criterion, while *n* is the sample size.

	**Coefficient**	**SE**	***p***		**Coefficient**	**SE**	***p***
T. retortaeformis: IOI				*G. strigosum*: IOI			
Intercept	7.89	0.325	<0.0001	Intercept	5.05	0.226	<0.0001
DPI	-3.48×10^{-2}	5.25×10^{-3}	<0.0001	DPI	-5.23×10^{-4}	3.20×10^{-3}	0.870
SI/DU	-0.516	0.503	0.305	SI/DU	-1.16×10^{-2}	0.290	0.968
DPI*SI/DU	2.62×10^{-3}	7.94×10^{-3}	0.742	DPI*SI/DU	6.78×10^{-3}	4.26×10^{-3}	0.111
AIC	949			*AIC*	674		
n	68			*n*	56		
T. retortaeformis: IL4				*G. strigosum*: IL4			
Intercept	2.61	0.462	<0.0001	Intercept	27.7	11.5	0.0193
DPI	0.0169	0.00744	0.0266	DPI	-0.154	0.162	0.348
SI/DU	2.12	0.714	0.00421	SI/DU	8.51	14.7	0.566
DPI*SI/DU	-0.0343	0.0112	0.00333	DPI*SI/DU	0.201	0.216	0.358
AIC	269			*AIC*	539		
n	68			*n*	56		
