# Supplementary material for: Within-host mechanisms of immune regulation explain the contrasting dynamics of two helminth species in both single and dual infections
Source: PLoS Comput Biol. 2020 Nov 23;16(11):e1008438. doi: 10.1371/journal.pcbi.1008438 (PMC7721179; doi:10.1371/journal.pcbi.1008438)
Supplement: S2 Table — AIC represents the Akaike Information Criterion, while n is the sample size. (PDF) [file pcbi.1008438.s005.pdf]

S2 Table. Generalized Linear Model (GLM) comparing the simulated intensity of infection (IOI, assuming a negative binomial distribution with a logarithmic link) or IL4 (assuming a normal distribution) by sampling time (days post-infection, DPI, as continous variable) and single/dual infection (SI/DU, as categorical variable), for *T. retortaeformis* and *G. strigosum*. *AIC* represents the Akaike Information Criterion, while *n* is the sample size.

	**Coefficient**	**SE**	***p***		**Coefficient**	**SE**	***p***
T. retortaeformis: IOI				*G. strigosum*: IOI			
Intercept	8.30	0.114	<0.0001	Intercept	5.08	6.85×10^{-2}	<0.0001
DPI	-5.98×10^{-2}	2.12×10^{-3}	<0.0001	DPI	-3.84×10^{-3}	9.86×10^{-4}	<0.0001
SI/DU	0.738	0.199	2.15×10^{-4}	SI/DU	-0.324	8.83×10^{-2}	2.49×10^{-4}
DPI*SI/DU	-4.42×10^{-2}	4.89×10^{-3}	<0.0001	DPI*SI/DU	7.00×10^{-3}	1.30×10^{-3}	<0.0001
AIC	772			*AIC*	519		
n	68			*n*	56		
T. retortaeformis: IL4				*G. strigosum*: IL4			
Intercept	2.95	0.226	<0.0001	Intercept	9.07	1.02	<0.0001
DPI	0.00157	0.00365	0.668	DPI	0.0206	0.0144	0.159
SI/DU	0.279	0.350	0.429	SI/DU	-3.55	1.31	8.88×10^{-3}
DPI*SI/DU	-0.0105	0.0055	0.0620	DPI*SI/DU	0.0625	0.0192	1.97×10^{-3}
AIC	172			*AIC*	268		
n	68			*n*	56		
